# Supplementary material for: The illusion of polygenicity in pool-seq genetic mapping studies: insufficient power can mask simple genetic architectures
Source: Genetics. 2026 Mar 11;233(1):iyag068. doi: 10.1093/genetics/iyag068 (PMC13147526; doi:10.1093/genetics/iyag068)
Supplement: iyag068_Supplementary_Data [file iyag068_supplementary_data.docx]

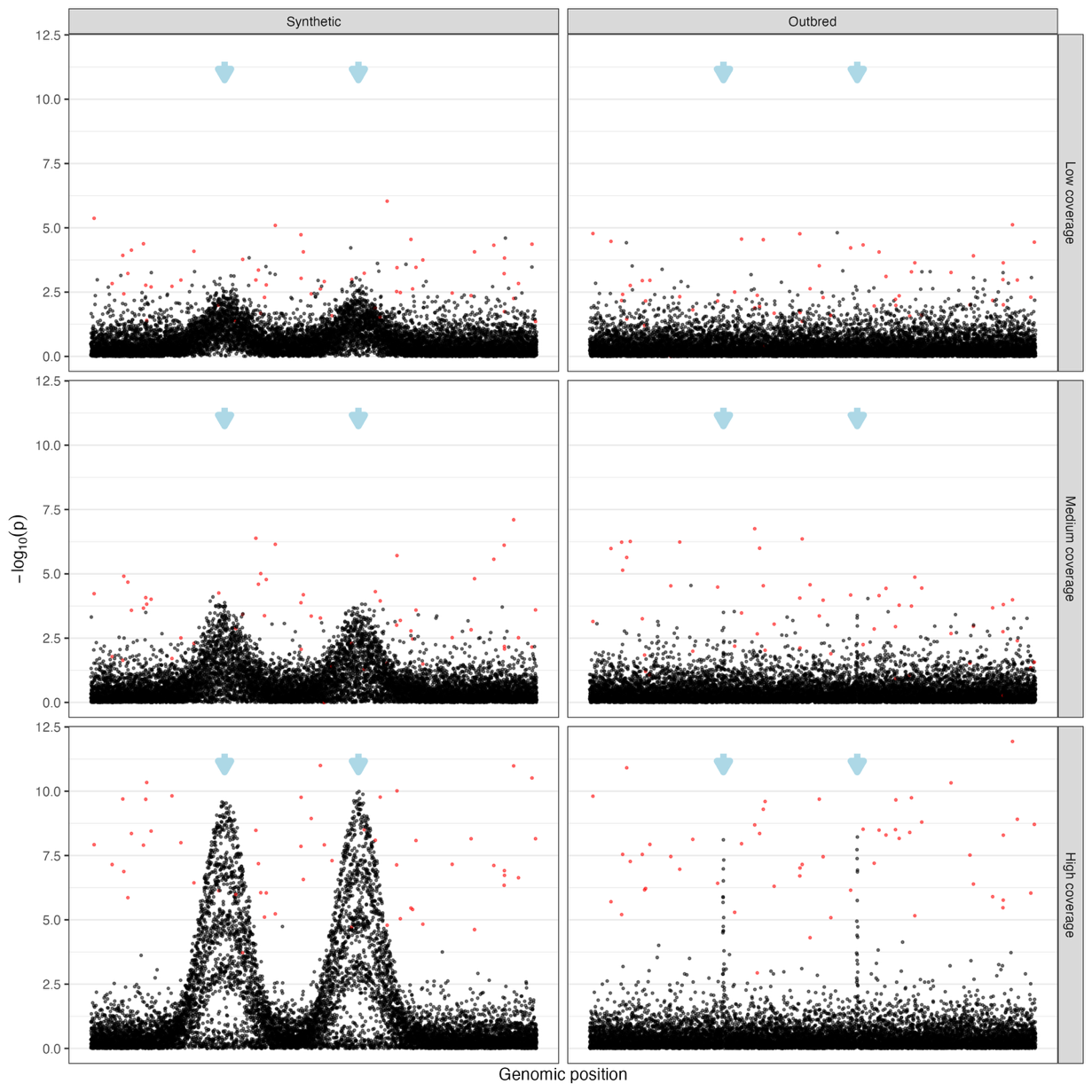


Supplementary Figure 1: An illustration of how true signals can be distinguished from alignment artifacts in pool-seq GWAS as a function of sequencing coverage and the scale of linkage disequilibrium. The panels depict Manhattan plots arranged by population type in columns (high LD synthetic populations, left; low LD natural populations, right) and sequencing coverage in rows. Points are tests for difference in allele frequency for directly ascertained SNPs. Black points are SNPs whose frequencies are accurately ascertained, whereas red points are SNPs prone to bioinformatic artifacts such as misalignment which result in false positive -log10(p) values. Light blue arrows mark the locations of two causal loci. High LD populations produce broad peaks of association with thousands of SNPs exhibiting signal over Mb sized windows, whereas low LD populations yield highly localized peaks marked by a handful of SNPs. Artifacts appear more significant at higher coverage but manifest as isolated, dispersed hits rather than clustered peaks, enabling them to be easily distinguished from true associations in high LD populations. This distinction is more challenging in low LD populations, especially at modest sequencing coverage, but is also highly depended on the precise scale of LD.


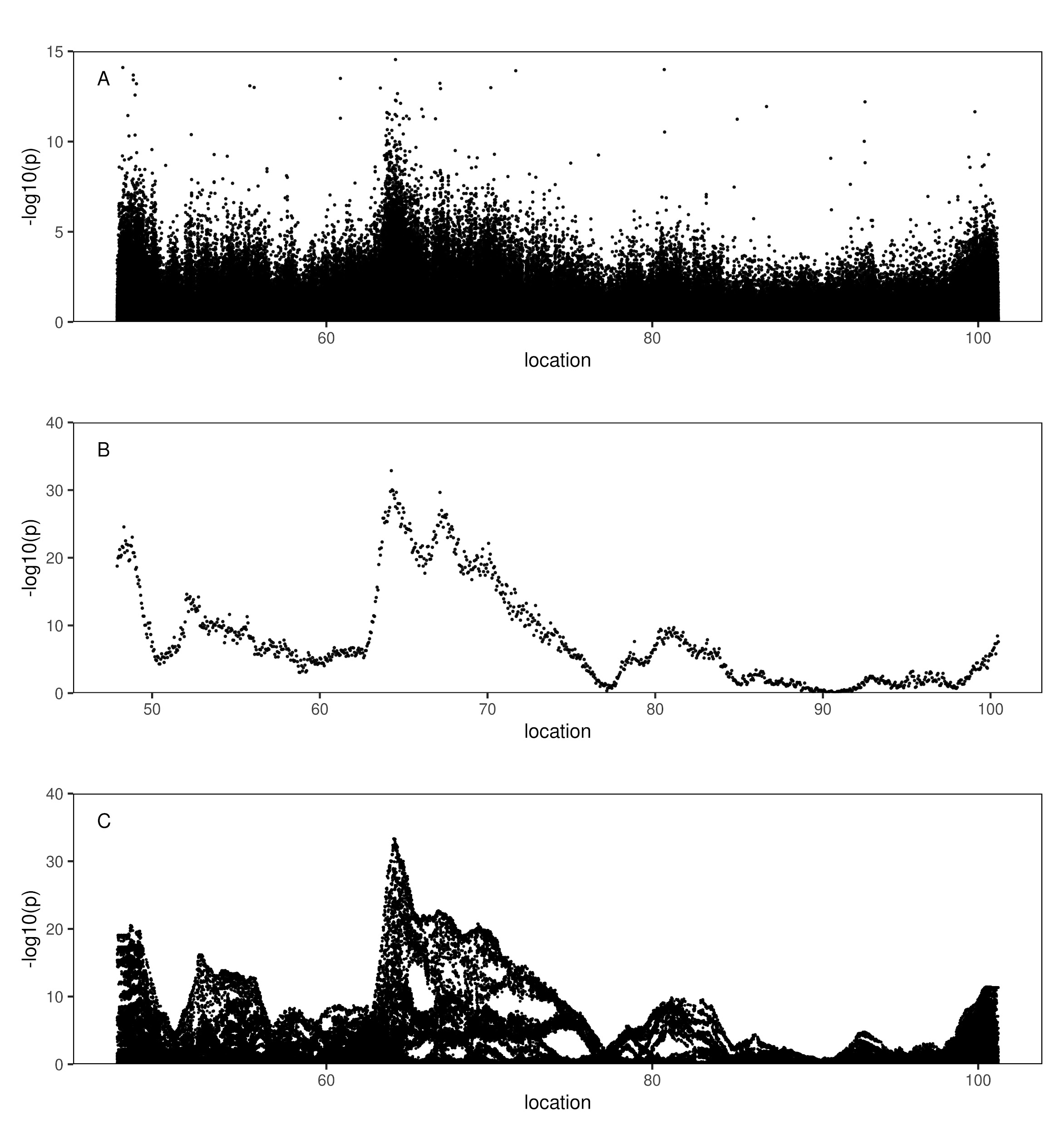


Supplementary Figure 2: Zinc chloride developmental resistance Manhattan plots associated with three different approaches to statistical testing, zoomed in to focus on chromosome 3R only: (A) directly ascertained REF and ALT SNP counts, (B) Imputed founder haplotype counts, and (C) imputed SNP counts. The panels are the same as Figure 3, except for panel A where we limit the Y-axis to 15.
